# Fatal Outcome of Intravenous Thrombolysis With an Unexpected Finding of Amyloid‐β‐Related Angiitis—A Case Report Highlighting a Relevant Scenario With Acute Focal Neurological Deficits and Minimal Radiological Presentation

**DOI:** 10.1111/neup.70013

**Published:** 2025-06-05

**Authors:** Kristof Babarczy, Bence L. Radics, Orsolya Horvath, Peter Klivenyi, Levente Szalardy

**Affiliations:** ^1^ Department of Neurology, Albert Szent‐Györgyi Medical School, Albert Szent‐Györgyi Clinical Center University of Szeged Szeged Hungary; ^2^ Institute of Pathology, Albert Szent‐Györgyi Medical School, Albert Szent‐Györgyi Clinical Center University of Szeged Szeged Hungary; ^3^ HUN‐REN‐SZTE Neuroscience Research Group Szeged Hungary

**Keywords:** amyloid spell, amyloid‐beta‐related angiitis, cerebral amyloid angiopathy, intracerebral hemorrhage, thrombolysis

## Abstract

Cerebral amyloid angiopathy (CAA) has been implicated as a risk for developing lobar intracerebral hemorrhage (ICH) after intravenous thrombolysis (IVT) applied for acute ischemic stroke (AIS). However, there is a paucity of cases reported with histopathological CAA diagnosis in this setting, with a single report to imply the role of CAA‐related inflammation (CAA‐RI). We report clinical, radiological, and neuropathological observations of a 65‐year‐old woman who presented with acute left‐hemispheric symptoms with an initially unrevealing cranial computed tomography (CT) and received IVT for presumed AIS. The course was rapidly complicated by a huge lobar ICH and a fatal outcome. The autopsy revealed severe CAA, unexpectedly with transmural CAA‐RI, a.k.a. amyloid‐β‐related angiitis (ABRA), and histopathological evidence for vascular amyloid‐β phagocytosis. Re‐evaluation of initial imaging did not reveal signs of asymmetric confluent white matter edema characteristic of CAA‐RI, but raised the suspicion of a tiny left central convexity subarachnoid hemorrhage, a substrate of amyloid spells. The genotype of the apolipoprotein E (ApoE) gene (*ApoE*) was ε3/ε3. Being the second published thrombolysis‐associated fatality with ABRA and among the few with definite CAA, the present case confirms CAA/CAA‐RI to be a potential hidden risk for IVT‐associated ICHs, urging for awareness of CAA‐associated pathologies and clinical‐radiological hints in an AIS setting. The findings implicate the relevance of vascular Aβ phagocytosis in the pathogenesis, confirm that CAA‐RI may present without prominent edema, highlight that CAA/CAA‐RI‐related focal neurological deficits (including amyloid spells) can be potential AIS mimics within the IVT time window, and urge for rigorous analysis of pre‐IVT CT scans for even subtle sulcal hyperdensities suggesting cSAH/amyloid spell in elderly patients, prompting consideration of magnetic resonance imaging.

**Abbreviations**
ABRA: Aβ‐related angiitis
AD: Alzheimer's disease
AIS: acute ischemic stroke
*ApoE*
: apolipoprotein E (ApoE) gene
Aβ: amyloid‐beta
CAA: cerebral amyloid angiopathy
CAA‐RI: CAA‐related inflammation
CMB: cerebral microbleed
CMT: Crossmon's modified Mallory's trichrome
cSAH: convexity subarachnoid hemorrhage
cSS: cortical superficial siderosis
CT: computed tomography
ICH: intracerebral hemorrhage
IVT: intravenous thrombolysis
MNGC: multinucleated giant cell
MRI: magnetic resonance imaging
WM: white matter

## 1 Introduction

Intravenous thrombolysis (IVT) in acute ischemic stroke (AIS) is relatively safe, with a 3%–8% risk for symptomatic and ~2% for fatal intracerebral hemorrhage (ICH). The decision‐making on IVT is generally based on computed tomography (CT) as the most time‐efficient modality. However, CT cannot detect chronic hemorrhagic alterations, including cerebral microbleeds (CMBs) and cortical superficial siderosis (cSS), which indicate conditions with potential risk for IVT‐ICHs, such as cerebral amyloid angiopathy (CAA) [1, 2, 3]. These can be visualized by hemosiderin‐sensitive magnetic resonance imaging (MRI) sequences, such as T2*‐gradient echo (GRE) and susceptibility‐weighted imaging (SWI).

Sporadic CAA is characterized by amyloid‐β (Aβ) deposition primarily in leptomeningeal/cortical arterioles, affecting up to 25% of the elderly at moderate‐to‐severe degree [3]. Alzheimer's disease (AD) is frequently concomitant, with the ε4 allele of apolipoprotein E (ApoE) gene (*ApoE*) being considered a mutual risk. Hallmark MRI features are strictly lobar hemorrhages, including CMB, convexity subarachnoid hemorrhage (cSAH), cSS, and ICH, enabling *probable CAA* diagnosis [1]. Typical symptoms include ICH‐associated signs, dementia, and transient focal neurological episodes characteristically due to corresponding cSAH/cSS (a.k.a. “amyloid spells”) [4]. Additionally, CAA can rarely manifest in subacute encephalopathy in a clinical‐radiological syndrome termed *CAA‐related inflammation* (CAA‐RI). Two histopathological subtypes are distinguished: (1) transmural CAA‐RI (a.k.a. Aβ‐related angiitis (ABRA) or vasculitic CAA‐RI) and (2) perivascular CAA‐RI (a.k.a. non‐vasculitic CAA‐RI), which are characterized by transmural and perivascular‐only lymphomonocytic inflammatory infiltration of CAA vessels, respectively, with or without multinucleated giant cells (MNGCs) [5]. Irrespective of histopathological subtype, radiologically CAA‐RI typically presents with asymmetric confluent white matter (WM) edema accompanying CAA‐compatible hemorrhages (*probable CAA‐RI*). Recently, leptomeningeal enhancement and sulcal non‐nulling on FLAIR were proposed to be incorporated into the criteria as alternative CAA‐RI presentations [5, 6].

Here, we present a case of a fatal IVT‐associated ICH, with an unexpected neuropathological finding of ABRA, the second in the literature. The detailed neuropathological analysis highlighted the role of phagocytosis of vascular Aβ in the pathogenesis. The CARE guidelines were followed.

## 2 Clinical Summary

The 65‐year‐old woman was admitted with a 45‐min history of sudden‐onset left‐hemispheric symptoms (right‐sided tingling, soon followed by right‐sided weakness and slurred speech; the time‐line of the clinical course is presented in Supporting Information, File 1). Her history included mild hypertension and insulin resistance, requiring no medication after lifestyle change. She experienced subjective memory impairment in the preceding months, and dull headache in the days before admission, felt similar to her habitual headaches. She suffered no head trauma. The family history included dementia (mother) and a disabling stroke (aunt).

**FIGURE 1:**
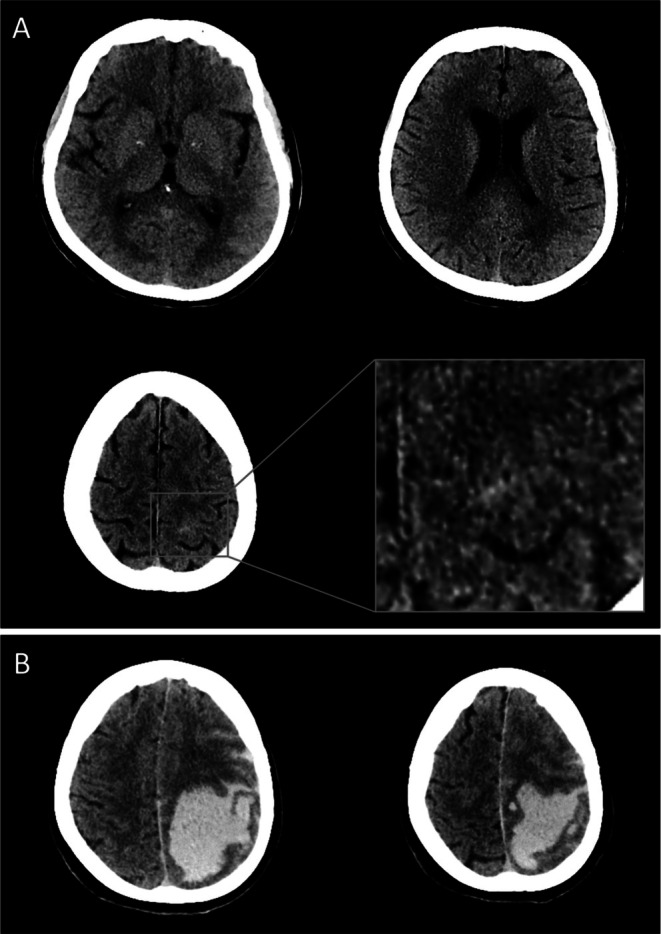
Radiological presentation. Initial cranial CT scan demonstrates moderate periventricular and deep white matter lucencies indicative of small vessel disease (A), with a tiny sulcal hyperdensity noted during retrospective evaluation (inlet of A). Urgent follow‐up CT scan after IVT demonstrates a large frontal–parietal ICH with subarachnoid extensions and finger‐like projections (B).

The neurological examination revealed dysarthria, right‐sided central facial palsy, moderate hemiparesis, hemiparesthesia, hemihypesthesia, and hyperreflexia. The blood pressure was 157/101 mmHg. Blood glucose and clotting parameters were normal. A cranial/carotid CT angiography was unrevealing (Figure 1A). Without evident contraindication and assuming AIS, IVT was initiated, 80 min after onset, with a 34‐min door‐to‐needle time. Ten minutes later, she had a headache, and at 20 min, she vomited and developed somnolence, gaze deviation to the left, global aphasia, and hemiplegia. Alteplase was stopped. An urgent CT scan demonstrated a huge left frontal–parietal ICH, with subarachnoid extensions and finger‐like projections (Figure 1B). The blood pressure was constantly within the recommended range. Despite osmotic therapy, the patient rapidly developed a coma and required mechanical ventilation. A third CT scan revealed enlarged ICH, edema, and tonsillar herniation. Repeated neurosurgical consultations did not recommend surgery. Two days later, brain death was determined.

Due to the surprisingly progressive IVT‐associated ICH morphologically resembling CAA‐related ICHs (simplified Edinburgh criteria: *CAA high probability* [7]), a re‐evaluation of initial imaging was performed for amyloid‐related abnormalities. No asymmetric edema was noted; however, a subtle sulcal hyperdensity was suspected left centrally, raising the suspicion of cSAH (Figure 1A). Neuropathological and molecular genetic work‐up was initiated.

## 3 Pathological Findings and Molecular Genetic Findings

After fixation in formaldehyde for 2.5 weeks, the brain weighed 1350 g. One‐mm‐thick subarachnoid hemorrhage was observed overlying the left hemisphere. Both hemispheres were swollen. In the deep WM of the left middle frontal gyrus, fresh colliquation was observed (30 × 20 × 20 mm). Dorsally to this, a blood‐filled cavity was seen (90 × 85 × 65 mm). The hippocampi were notable for slight meningeal thickening. The basal ganglia were unremarkable. The cerebellum and brainstem showed congestion with secondary stripe‐like hemorrhages. Methods related to histopathology are summarized in Supporting Information, File 2.

**FIGURE 2:**
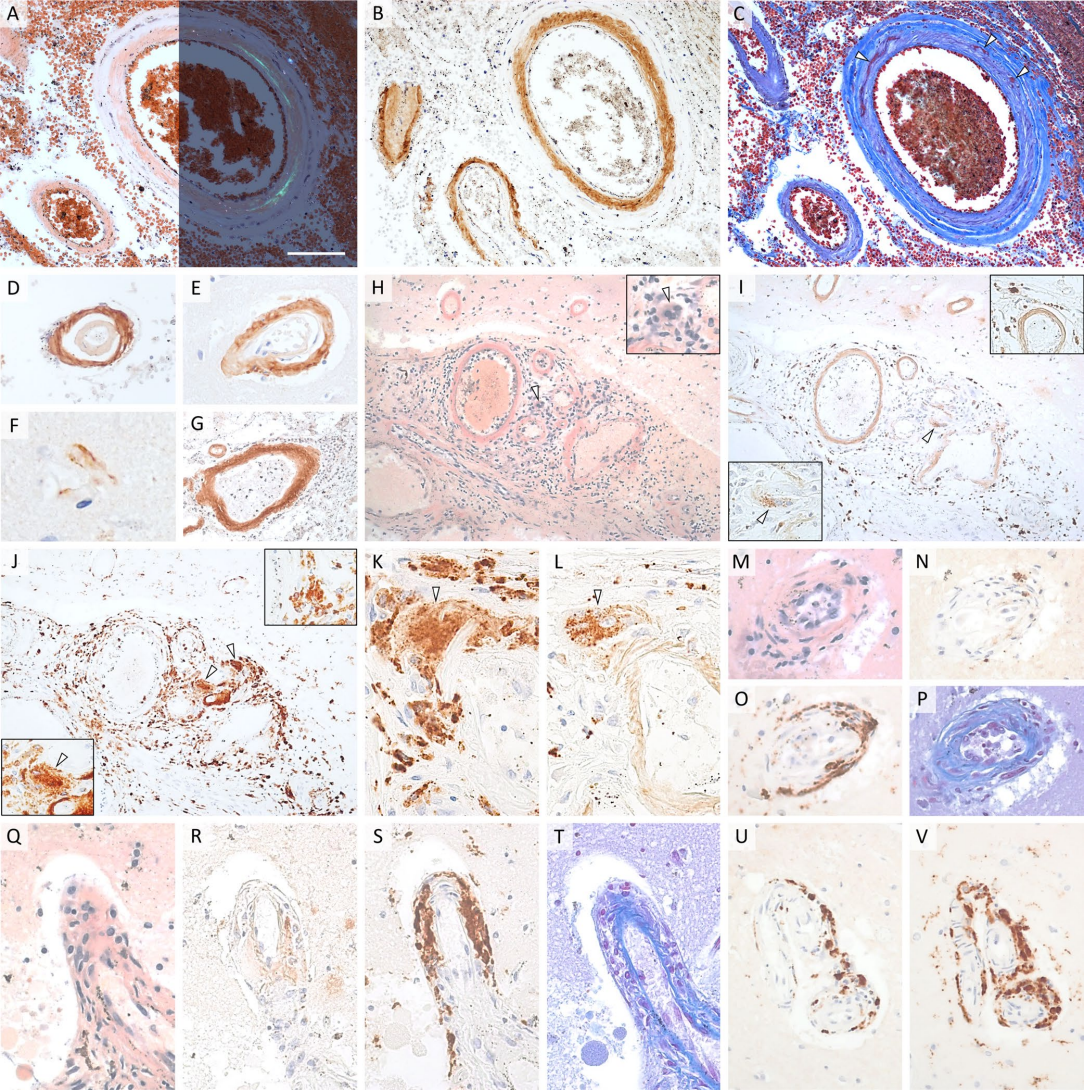
Neuropathological presentation. Frontal vessels near the ICH display congophilic CAA, with apple‐green birefringence under the polarized microscope (A) and Aβ immunopositivity (B). Fibrinoid necrosis (Vonsattel grade 4, C). Double‐barrel formations in the leptomeninges (D) and parenchyma (E). Capillary involvement (Thal CAA Type 1, F). A single vessel shows perivascular mononuclear cuffing in this region (G). Massive inflammatory infiltrate encompasses CAA vessels in the collateral sulcus and adjacent cortices (H–V), comprising several lymphocytes, with abundant CD68‐positive microglia/macrophages (J, K, O, S, V). Many cells contain intracytoplasmic Aβ (I, L, N, U). MNGCs (arrows in H–L), often with intracytoplasmic Aβ (arrows in I and L). Intramural involvement (M–V), consistent with ABRA. Remarkably paler vascular Aβ positivity in the inflamed area (I, L, R) compared to frontal CAA (B, D, E, G). Vessels with the most severe vasculitis demonstrate extremely pale (R) or minimal‐to‐no vascular Aβ positivity (N, U), but are associated with various amount of cells with intracytoplasmic Aβ (N, U), occasionally in a pattern almost indistinguishable from CD68 positivity (U, V), reflecting phagocytosis of vascular Aβ. Congo red: (A), (H), (M), (Q); anti‐Aβ antibody: (B), (D–G), (I), (L), (N), (R), (U); Crossmon's modified Mallory's trichrome: (C), (P), (T); anti‐CD68 antibody: (J, K), (O), (S), (V). Scale bar in (A) represents 100 μm in (A–C), 65 μm in (D, M–P, U, V), 55 μm in (E, Q–T), 20 μm in (F), 145 μm in (G–J), and 35 μm in (K, L).

Surrounding the frontal ICH margin, leptomeningeal/cortical vessels contained congophilic amyloid with apple‐green birefringence, confirmed to be Aβ, consistent with CAA (Figure 2A,B). The double‐barrel formations and extensive fibrinoid necrosis rendered the severity to be Vonsattel grade 4 (Figure 2C–E); the extension was Love grade 2 and 3 in the parenchyma and leptomeninges, respectively. The capillary involvement yielded Thal CAA Type 1 (Figure 2F). The small emollition in the middle frontal gyrus was consistent with acute infarct; however, the ICH impeded the etiological evaluation of territories corresponding to the initial symptoms. No evident ruptured vessels [8] or Prussian blue‐positive siderophages indicating prior hemorrhage were noted. The erythrocyte‐filled subarachnoid space contained slight non‐specific mononuclear inflammatory infiltration not associated with vessels, with only 1 CAA vessel demonstrating perivascular cuffing (Figure 2G).

In the right collateral sulcus, many leptomeningeal CAA vessels and some of their perforators were associated with overt perivascular and transmural inflammation, comprising predominantly CD4‐positive and CD8‐positive T lymphocytes, few CD20‐positive B lymphocytes, and abundant CD68‐positive microglia/macrophages, consistent with ABRA (Figure 2H–V, Supporting Information, File 3). Many inflammatory cells showed intracytoplasmic Aβ positivity, including perivascular MNGCs (identified as CD68+ macrophages), indicating phagocytosis (Figure 2I–L). Notably, vascular Aβ positivity here was remarkably pale. In fact, the most severely vasculitic vessels showed minimal‐to‐no Aβ positivity but were associated with perivascular/intramural cells with intracytoplasmic Aβ, occasionally in a pattern almost indistinguishable from CD68 positivity, suggesting phagocytosis of vascular Aβ (Figure 2N,R,U,V). The subarachnoid infiltrate also contained a few scattered erythrocytes with occasional single siderophages suggesting erythrocyte extravasation but no overt hemorrhage. Numerous diffuse and a few dense core Aβ plaques were noted but with only occasional neurofibrillary tangles in the entorhinal cortex but not in the hippocampus (Braak stage 1/2), excluding AD. The hippocampus proper was affected by mild‐to‐moderate CAA. The deep subcortical WM was devoid of CAA. The basal ganglia showed mild arteriosclerosis (with occasional single perivascular siderophages) but no CAA.

Restriction fragment length polymorphism analysis of *ApoE* revealed an ε3/ε3 genotype.

## 4 Discussion

This is the second report on the sequelae of IVT applied for suspected AIS in a patient with ABRA at autopsy, preceded by a report on a 55‐year‐old man with multiple IVT‐ICHs, likewise without clinical‐radiological hints of CAA‐RI [9].

CAA‐RI (including its vasculitic subtype, ABRA) is a rare inflammatory CAA manifestation, typically presenting with cognitive/behavioral changes, headache, focal neurological deficits, and/or seizures, predominantly in association with asymmetric confluent WM edema and CAA‐compatible hemorrhagic alterations. However, alternative (strictly leptomeningeal) presentations have been highlighted in recent extended criteria [5, 6]. Most recently, a study found association between cSAH‐related spells and corresponding leptomeningeal enhancement in cases without WM edema, suggesting a causal link between cSAH and leptomeningeal CAA‐RI [10]. The diagnostic relevance of CAA‐RI is established by its propensity to respond to immunosuppression (> 80%). Without MRI, it is unclear whether our patient would have met *probable CAA‐RI* by existing criteria; however, apparent confluent edema was absent. Though CAA was suspected based on ICH morphology, CAA‐RI was not suspected until histopathological work‐up. Speculatively, the recent memory complaints and headache might be discussed as correlates of CAA‐RI, but these were not bothersome enough to prompt seeking medical help. The mean 10 years earlier presentation of CAA‐RI (67 years) compared to that of the first CAA‐related ICH fits with the age of our case [2, 5]. The majority of CAA‐RI cases have *ApoE‐*ε4/ε4 genotype [5], which was, however, not present.

The risk for lobar ICHs posed by CAA, especially with concomitant antithrombotics, has long been recognized [2]. Additionally, increasing evidence links IVT‐ICHs to CAA. Early clinical evidence linking pathology‐verified CAA to IVT‐ICHs came from acute myocardial infarction cases; however, the concomitant heparinization obscured the interpretation [11]. More recently, three cases suffering ICH(s) after IVT used for AIS have been reported with definite (autopsy‐verified) CAA, 1 with ABRA (Supporting Information, File 4) [9, 12, 13]. Systematically, a 13.3% (2/15) prevalence of symptomatic IVT‐ICHs was found in AIS cases with *probable CAA* on pretreatment MRI (higher than in unselected populations), with invariable fatality [14]. Similarly, 53.8% (7/13) of AIS cases with IVT‐associated lobar remote parenchymal hemorrhage had strictly lobar (CAA‐compatible) CMBs on post‐treatment MRI, versus 3.0% in those without hemorrhage [15]. Correspondingly, significantly higher cortical Pittsburgh compound B retention was reported in patients with versus those without parenchymal hemorrhage after IVT [16]. Systematic autopsy data are lacking.

Though an increased risk for hemorrhage formation posed by CAA‐RI in particular has only recently been supported by systematic analysis [5], a medicolegal autopsy case was reported with ABRA as a proposed etiology underlying fatal multiple lobar spontaneous ICHs [17]. Similarly to our case, the authors reported the paucity of Aβ in severely vasculitic vessels and the presence of phagocytosed Aβ in MNGCs [17]. Phagocytosed Aβ in microglia, macrophages, or MNGCs is frequently described in CAA‐RI (reviewed in [5, 18]). Remarkably, this seemingly inverse relationship between vascular Aβ load and vasculitic severity has also been noted by a few reports [5, 17, 19], most of them also observing phagocytosed Aβ [5, 17], in the above case with concomitant ICH [17]. These together with our corroborating observations imply a possible role of phagocytic Aβ clearance in hemorrhage formation in CAA‐RI.

Our case expands the knowledge on the potential complications of CAA/CAA‐RI; however, the etiology underlying her initial symptoms is a dilemma, as faced before [13]. Indeed, the ICH‐associated destruction precluded the verification of primary ischemia. Additionally, CAA can present with “amyloid spell”, often with corresponding cSAH. From this respect, we cannot exclude that the identified vague sulcal hyperdensity had etiological relevance, especially in light of the recently proposed link between cSAH and leptomeningeal CAA‐RI [10]; however, it was not considered hemorrhage by radiologists/neurologists during acute care, and might have required confirmatory MRI. Finally, the symptoms could be related to CAA‐RI itself.

This case study has several learning points and peculiarities: (a) it confirms CAA/CAA‐RI to be a risk for IVT‐ICH, implying the additive role of inflammation; (b) reports the second ABRA case in this scenario; (c) strongly implicates the role of vascular Aβ phagocytosis; (d) confirms that CAA‐RI may present without prominent edema; (e) highlights that CAA/CAA‐RI‐related focal deficits (including amyloid spells) can be potential AIS mimics within the IVT time window, suggesting their consideration in the differential diagnosis; and (f) urges for rigorous analysis of pre‐IVT CT scans for even subtle sulcal hyperdensities suggesting cSAH/amyloid spell in elderly patients, prompting consideration of MRI at the slightest suspicion.

## Disclosure

L.S. received speaker's honoraria from Biogen and conference registration fees from Bayer, Biogen, Gedeon Richter Plc., Sandoz, and Sanofi‐Genzyme.

## Ethics Statement

The study and the publication of the case were approved by local Ethical Committee (44/2016, 22/2021).

## Consent

The next‐of‐kin provided informed consent to the genetic analysis and publication.

## Conflicts of Interest

The authors declare no conflicts of interest.

## Supporting information


**Figure S1.** The time course of clinical signs and symptoms in relation to the intravenous thrombolysis.

**Table S1.** Antibodies used for immunohistochemistry.

**Figure S2.** Immunohistochemical characterization of small round cells in the infiltrate surrounding CAA vessels in the collateral sulcus.

**Table S2.** Individual reported cases with IVT‐associated ICH and pathological evidence of CAA.

## Data Availability

The data that support the findings of this study are available from the corresponding author upon reasonable request.
