# Supplementary material for: Fatal Outcome of Intravenous Thrombolysis With an Unexpected Finding of Amyloid‐β‐Related Angiitis—A Case Report Highlighting a Relevant Scenario With Acute Focal Neurological Deficits and Minimal Radiological Presentation
Source: Neuropathology. 2025 Jun 5;45(4):e70013. doi: 10.1111/neup.70013 (PMC12279614; doi:10.1111/neup.70013)
Supplement: Supplementary file 1 — Figure S1. The time course of clinical signs and symptoms in relation to the intravenous thrombolysis. [file NEUP-45-0-s001.docx]

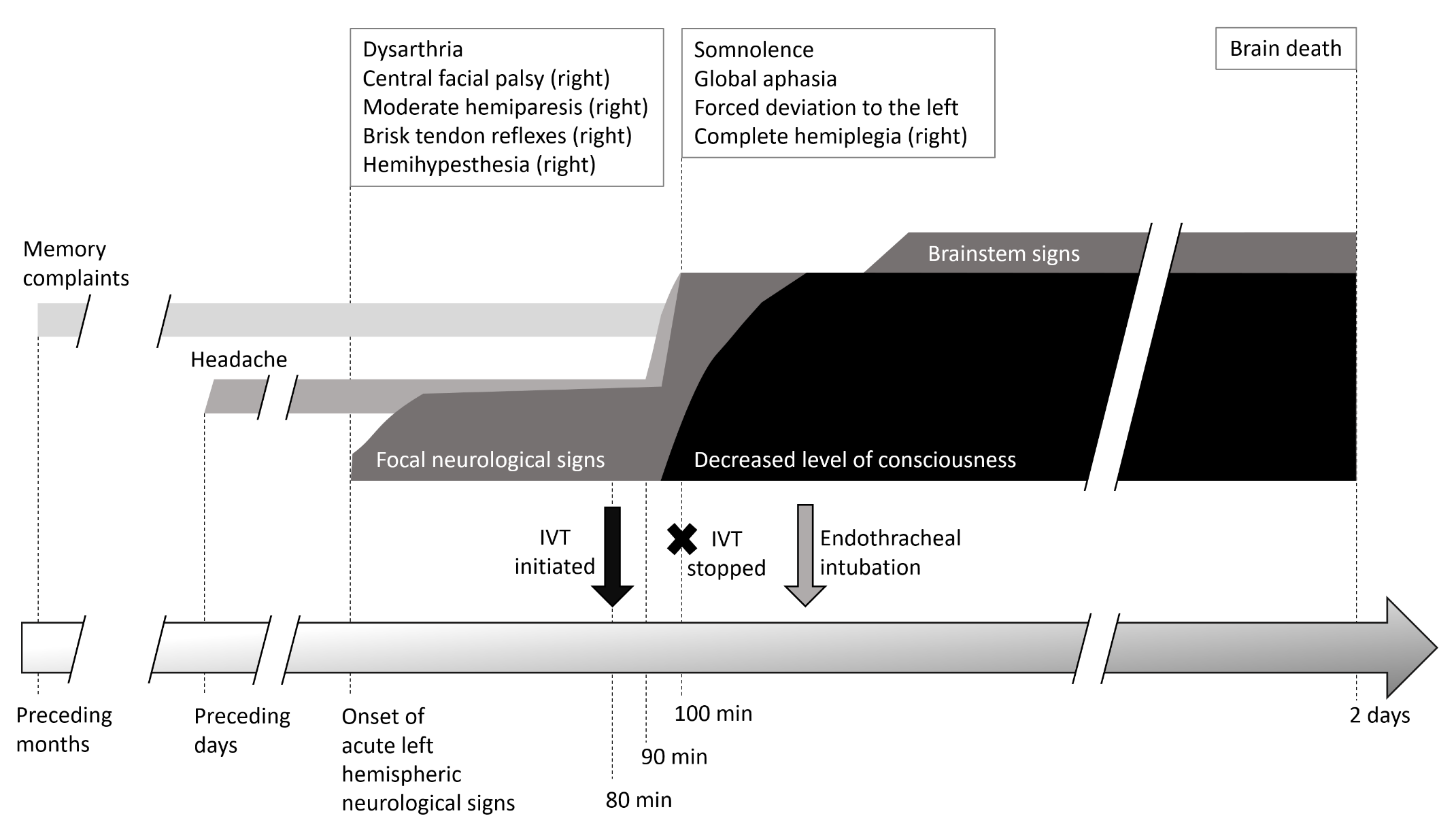


**Supplementary Fig 1** The time course of clinical signs and symptoms in relation to the intravenous thrombolysis.

IVT, intravenous thrombolysis.
