# Supplementary material for: Fatal Outcome of Intravenous Thrombolysis With an Unexpected Finding of Amyloid‐β‐Related Angiitis—A Case Report Highlighting a Relevant Scenario With Acute Focal Neurological Deficits and Minimal Radiological Presentation
Source: Neuropathology. 2025 Jun 5;45(4):e70013. doi: 10.1111/neup.70013 (PMC12279614; doi:10.1111/neup.70013)
Supplement: Supplementary file 2 — Table S1. Antibodies used for immunohistochemistry. [file NEUP-45-0-s002.docx]

**MATERIALS AND METHODS OF HISTOPATHOLOGY**

5-μm-thick sections of formalin-fixed paraffin-embedded brain tissue blocks were used for histopathology from the following territories: left superior frontal gyrus containing the anterior edge of the intracerebral hemorrhage (ICH), left middle frontal gyrus anterior to the ICH, right superior and middle temporal gyri, right anterior hippocampus, right posterior hippocampus, and left basal ganglia at the level of the lentiform nucleus. In addition to hematoxylin and eosin staining, Congo red, Prussian blue, and Crossmon’s modified Mallory’s trichrome stainings were performed to visualize amyloid deposition, hemosiderin deposition, and fibrinoid change, respectively. Immunohistochemistry (IHC) was performed on Leica BOND-MAX fully automated IHC staining system applying the following antibodies and parameters (**Supplementary table 1**).

**Supplementary table 1** Antibodies used for immunohistochemistry

| Antibody | Clone | Host | Type | Manufacturer | Dilution | ER mode | Incubation time |
| --- | --- | --- | --- | --- | --- | --- | --- |
| CD68 | PG-M1 | mouse | monoclonal | DAKO | 1:100 | ER2 (pH 9.0) | 20 min |
| CD4 | 4B12 | mouse | monoclonal | DAKO | RTU (1:1) | ER2 (pH 9.0) | 20 min |
| CD8 | C8/144B | mouse | monoclonal | DAKO | RTU (1:1) | ER2 (pH 9.0) | 20 min |
| CD20 | L26 | mouse | monoclonal | Genemed | 1:200 | ER2 (pH 9.0) | 20 min |
| Aβ | RBT-A4 | rabbit | monoclonal | BioSB | 1:400 | ER2 (pH 9.0) | 20 min |
| Tau | RB-1429 | rabbit | polyclonal | Thermo Fisher | 1:400 | ER1 (pH 6.0) | 30 min |

Aβ, Amyloid-beta; ER, epitope retrieval; RTU, ready-to-use.
