# Supplementary material for: Fatal Outcome of Intravenous Thrombolysis With an Unexpected Finding of Amyloid‐β‐Related Angiitis—A Case Report Highlighting a Relevant Scenario With Acute Focal Neurological Deficits and Minimal Radiological Presentation
Source: Neuropathology. 2025 Jun 5;45(4):e70013. doi: 10.1111/neup.70013 (PMC12279614; doi:10.1111/neup.70013)
Supplement: Supplementary file 3 — Figure S2. Immunohistochemical characterization of small round cells in the infiltrate surrounding CAA vessels in the collateral sulcus. [file NEUP-45-0-s004.docx]

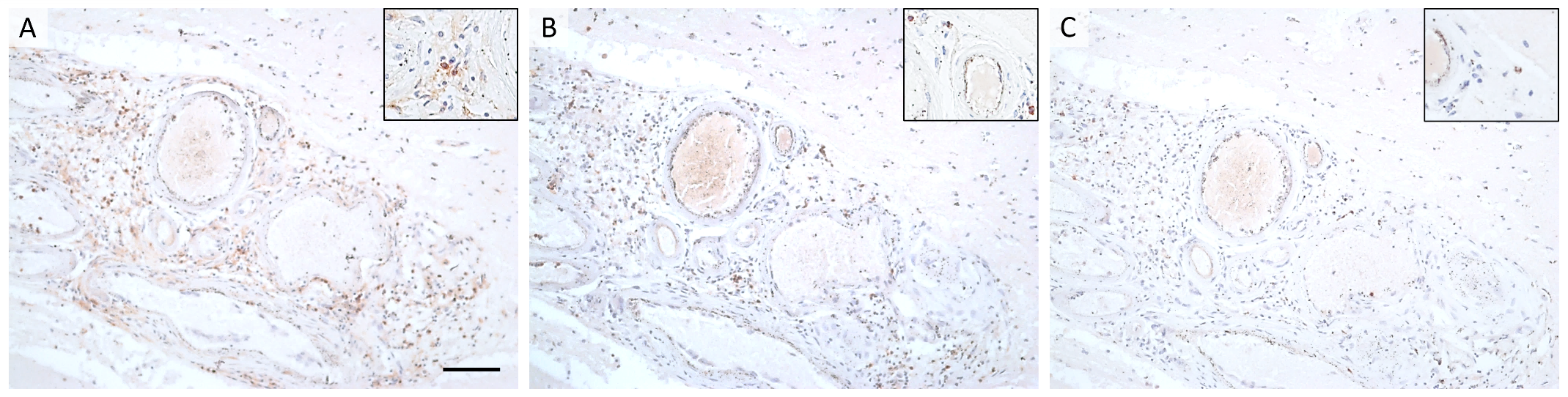


**Supplementary Fig 2** Immunohistochemical characterization of small round cells in the infiltrate surrounding CAA vessels in the collateral sulcus, demonstrating many helper T (anti-CD4 antibody; **A**), several cytotoxic T (anti-CD8 antibody, **B**), and a few B lymphocytes (anti-CD20 antibody; **M**).

Scale bar in (**A**) represents 100 μm in (**A**-**C**).
