# Supplementary material for: Fatal Outcome of Intravenous Thrombolysis With an Unexpected Finding of Amyloid‐β‐Related Angiitis—A Case Report Highlighting a Relevant Scenario With Acute Focal Neurological Deficits and Minimal Radiological Presentation
Source: Neuropathology. 2025 Jun 5;45(4):e70013. doi: 10.1111/neup.70013 (PMC12279614; doi:10.1111/neup.70013)
Supplement: Supplementary file 4 — Table S2. Individual reported cases with IVT‐associated ICH and pathological evidence of CAA. [file NEUP-45-0-s003.docx]

**Supplementary table 2** Individual reported cases with IVT-associated ICH and pathological evidence of CAA

| 1^st^ author  (case no.)^ref.^ | age (y) | sex | IVT ind. | IVT agent | con. hep. | ICH onset | lobar ICH site ± other H types | time till death^†^ | spec. | CAA grade**^‡^** | Thal type**^§^** | inflam. on hist. |
| --- | --- | --- | --- | --- | --- | --- | --- | --- | --- | --- | --- | --- |
| Ramsay[^1^](#_heading=h.wpup8jp8snmq) | 56 | m | AMI | sk | 1^¶^ | 8 h | mult. L T-P +SAH+SDH | 5.5 d | aut. | mod. | 1 | none |
| Pendlebury[^2^](#_heading=h.8ez9cvq4n25b) | 60 | f | AMI | rtPA | 1 | 6 h | R F & L T +IVH+SAH | 32 h | aut. | mod. | n.a. | n.a. |
| Leblanc[^3^](#_heading=h.hnw7pk5aifgv) | 66 | m | AMI | sk | 1 | <18 h | R T+SAH | 5 d^††^ | sur. | mod. | n.a. | n.a. |
| Wijdicks[^4^](#_heading=h.8fdrcil1e1t) | 75 | m | AMI | sk | 1 | 12 h | mult.+IVH+SAH | 14 h | sur. | mod. | n.a. | n.a. |
| Sloan (6)[^5^](#_heading=h.4z0gs2o1arek) | 67 | n.a. | AMI | rtPA | 1 | 11.8 h | mult. | 21 d | aut. | mod.^‡‡^ | n.a. | n.a. |
| Sloan (15)[^5^](#_heading=h.4z0gs2o1arek) | 67 | n.a. | AMI | rtPA | 1 | 7.5 h | mult.+IVH | 3 d | aut. | mod.^‡‡^ | n.a. | n.a. |
| Sloan (21)[^5^](#_heading=h.4z0gs2o1arek) | 68 | n.a. | AMI | rtPA | 1 | 9.5 h | mult. | 2 d | aut. | mod.^‡‡^ | n.a. | n.a. |
| Felling (1)[^6^](#_heading=h.ykol8c7n9mp1) | 81 | f | AIS | rtPA | 0 | 1 h | mult.+IVH+SAH+SDH | n.a. | aut. | severe | n.a. | n.a. |
| Mattila[^7^](#_heading=h.jnh6lu729vp9) | 71 | f | AIS | rtPA | 0 | 1 h | mult.+SAH+SDH | 6 d | aut. | severe | n.a. | n.a. |
| Reisz[^8^](#_heading=h.92o9ihfhz2bz) | 55 | m | AIS | rtPA | 0 | <2 h | mult.+SAH+IVH | 4 d | aut. | severe | n.a. | ABRA |
| Present study | 65 | f | AIS | rtPA | 0 | 20 min | L F-P+IVH+SAH | 2 d | aut. | severe | 1 | ABRA |

ABRA, amyloid-beta-related angiitis; aut., autopsy; AIS, acute ischemic stroke; AMI, acute myocardial infarction; CR, Congo red; CAA, cerebral amyloid angiopathy, con., concomitant; d, day; f, female; F, frontal; h, hour; hep., heparin; hist., histology; ICH, intracerebral hemorrhage; ind., indication; inflam., inflammation; IVH, intraventricular hemorrhage; IVT, intravenous thrombolysis; L, left; m, male; min, minute; mod., moderate; mult., multiple; n.a., data not available; no., number; P, parietal; R, right; ref., reference; rtPA; recombinant tissue plasminogen activator; SAH, subarachnoid hemorrhage; SDH, subdural hemorrhage; sk, streptokinase; spec, specimen; sur., surgical, T, temporal; y, year; 0, not applied/not performed; 1, applied/performed.

^†^ after admission/therapy initiation

^‡^ retrospective identification of Vonsattel grade (1 = mild, 2 = moderate, 3-4 = severe) was performed based on published descriptions and/or images, reporting bias may apply

^§^ Type 1 and 2: with and without capillary involvement, respectively

^¶^ based on reported laboratory parameters

^††^ non-CAA-related cause (recurrent acute myocardial infarction)

^‡‡^ generalized description corresponds to Vonsattel grade 2 with extensive involvement in cases 6 and 21, and mild in case 15

**SUPPLEMENTARY REFERENCES**

1. Ramsay DA, Penswick JL, Robertson DM. Fatal streptokinase-induced intracerebral haemorrhage in cerebral amyloid angiopathy. *Can J Neurol Sci* 1990;**17**: 336-41.

2. Pendlebury WW, Iole ED, Tracy RP, Dill BA. Intracerebral hemorrhage related to cerebral amyloid angiopathy and t-PA treatment. *Ann Neurol* 1991;**29**: 210-3.

3. Leblanc R, Haddad G, Robitaille Y. Cerebral hemorrhage from amyloid angiopathy and coronary thrombolysis. *Neurosurgery* 1992;**31**: 586-90.

4. Wijdicks EF, Jack CR, Jr. Intracerebral hemorrhage after fibrinolytic therapy for acute myocardial infarction. *Stroke* 1993;**24**: 554-7.

5. Sloan MA, Price TR, Petito CK *et al*. Clinical features and pathogenesis of intracerebral hemorrhage after rt-PA and heparin therapy for acute myocardial infarction: the Thrombolysis in Myocardial Infarction (TIMI) II Pilot and Randomized Clinical Trial combined experience. *Neurology* 1995;**45**: 649-58.

6. Felling RJ, Faigle R, Ho CY, Llinas RH, Urrutia VC. Cerebral Amyloid Angiopathy: A Hidden Risk for IV Thrombolysis? *J Neurol Transl Neurosci* 2014;**2**.

7. Mattila OS, Sairanen T, Laakso E, Paetau A, Tanskanen M, Lindsberg PJ. Cerebral amyloid angiopathy related hemorrhage after stroke thrombolysis: case report and literature review. *Neuropathology* 2015;**35**: 70-4.

8. Reisz Z, Troakes C, Sztriha LK, Bodi I. Fatal thrombolysis-related intracerebral haemorrhage associated with amyloid-beta-related angiitis in a middle-aged patient - case report and literature review. *BMC Neurol* 2022;**22**: 500.
